# Impact of treatment response to neoadjuvant chemotherapy on brain metastasis patterns and breast cancer prognosis

**DOI:** 10.1016/j.breast.2025.104650

**Published:** 2025-11-14

**Authors:** Jihwan Yoo, Yoon Jin Cha, Sung Gwe Ahn, Joon Jeong, Hun Ho Park, Sung Jun Ahn, Bio Joo, Ji Hyun Park, Jee Hung Kim, Soong June Bae

**Affiliations:** a Department of Neurosurgery, Brain Tumor Center, Gangnam Severance Hospital, Yonsei University, College of Medicine, Seoul, Republic of Korea; b Department of Pathology, Gangnam Severance Hospital, Yonsei University, College of Medicine, Seoul, Republic of Korea; c Institute for Breast Cancer Precision Medicine, Yonsei University College of Medicine, Seoul, Republic of Korea; d Department of Surgery, Gangnam Severance Hospital, Yonsei University, College of Medicine, Seoul, Republic of Korea; e Department of Radiology, Gangnam Severance Hospital, Yonsei University, College of Medicine, Seoul, Republic of Korea; f Division of Medical Oncology, Department of Internal Medicine, Gangnam Severance Hospital, Yonsei University, College of Medicine, Seoul, Republic of Korea

**Keywords:** Breast cancer, Pathologic complete response, Triple-negative breast cancer, Brain metastasis

## Abstract

**Background:**

Brain metastases (BM) are a major cause of mortality in breast cancer. While pathologic complete response (pCR) after neoadjuvant chemotherapy is associated with favorable survival outcomes, its impact on BM development and prognosis remains unclear.

**Methods:**

We retrospectively analyzed 1,244 patients with early-stage breast cancer who underwent neoadjuvant chemotherapy followed by surgery. Clinicopathological features, BM incidence, and survival outcomes were assessed. Propensity score matching (PSM) was applied to adjust for baseline differences. Gene expression profiling was performed in BM samples from pCR and non-pCR patients.

**Results:**

Of these, 437 (35.1 %) patients achieved pCR and 52 (4.2 %) developed BM. In TNBC, non-pCR patients had a significantly higher BM rate (9.2 % vs. 3.3 %, *P* = 0.026), whereas no differences were observed in other subtypes. Patients with BM who achieved pCR were more likely to present with single brain lesion (42.9 % vs. 10.5 %, *P* = 0.016), undergo craniotomy (71.4 % vs. 31.6 %, *P* = 0.010), and less frequently had extracranial metastases (28.6 % vs. 73.7 %, *P* = 0.003). Median overall survival after BM was longer in the pCR (42 vs. 4 months, *P* = 0.002), and this benefit remained significant after PSM (43 vs. 10 months, *P* = 0.033). Transcriptomic analysis identified distinct molecular profiles, with upregulation of RPL27A and CTLA4 in pCR BM and non-pCR BM.

**Conclusions:**

pCR was associated with lower metastatic burden and improved survival following BM diagnosis. Molecular differences between pCR and non-pCR BM suggest distinct mechanisms of metastatic evolution, suggesting the need for tailored surveillance and preventive strategies.

## Highlights

• Brain metastases (BM) occurred in 4.2 % of 1,244 patients after neoadjuvant chemotherapy.
• Lower BM incidence with pCR was observed only in triple-negative breast cancer.
• Patients with pCR who developed BM had lower metastatic burden and longer survival than non-pCR patients.
• RPL27A and CTLA4 were upregulated in pCR-BM, whereas PLXND1 was enriched in non-pCR lesions.

## 1 Introduction

Breast cancer remains the leading cause of cancer-related mortality worldwide, and brain metastases (BM) pose a significant clinical challenge [1,2]. Human epidermal growth factor receptor 2 (HER2)-positive and triple-negative breast cancer (TNBC) are molecular breast cancer subtypes that exhibit higher BM propensities than hormone receptor (HR)-positive breast cancer [1,3]. Advancements in systemic treatments, including CDK4/6 inhibitors [4,5], HER2-targeted therapies [6,7], and immune checkpoint inhibitors [8] have improved survival outcomes for patients with metastatic breast cancer; however, the prognosis for patients with BM remains poor, highlighting the need for improved risk stratification and effective treatment strategies.

Neoadjuvant chemotherapy is increasingly used to downstage tumors and optimize adjuvant treatment strategies in patients with stage II or III breast cancer, particularly those with HER2-positive breast cancer and TNBC [9,10]. Pathologic complete response (pCR), defined as the absence of residual invasive cancer in both the breast and lymph nodes following neoadjuvant chemotherapy, is strongly associated with a favorable prognosis [11,12]. Reportedly, patients who achieve pCR have a lower incidence of distant metastases, likely due to the ability of neoadjuvant chemotherapy to significantly eradicate microscopic distant disease, consequently lowering the risk of distant metastasis [13]. However, the effect of treatment response to neoadjuvant chemotherapy on the development and prognosis of BM remains unclear. Understanding the relationship between the treatment response and BM characteristics is essential to optimize surveillance strategies and therapeutic interventions.

In this study, we aimed to evaluate the link between treatment response and the BM clinical features, as well as survival outcomes, in patients with breast cancer who received neoadjuvant chemotherapy. Furthermore, we aimed to identify potential biological mechanisms by conducting gene expression analysis to explore the molecular differences in BM cases depending on the response to neoadjuvant chemotherapy.

## 2 Materials and methods

### 2.1 Patients

This study was approved by the Institutional Review Board of the corresponding author's institution (IRB no. 3-2025-0083). The study followed the Good Clinical Practice guidelines and the principles of the Declaration of Helsinki. The requirement for informed consent was waived due to the retrospective study design.

**Supplementary Fig. 1:**
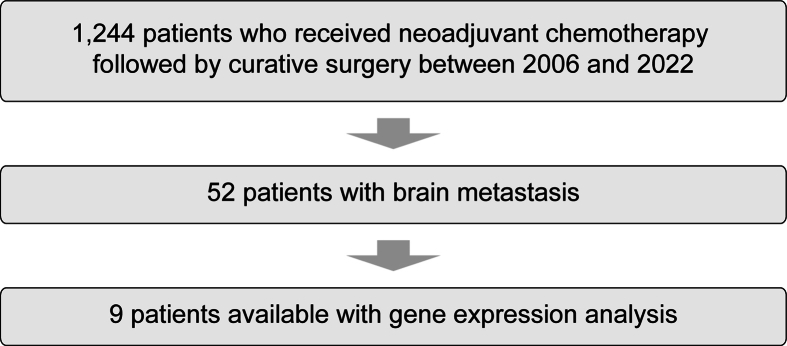
Study population.

A consortium diagram of the study population is shown in Supplementary Fig. 1. We retrospectively identified 1,244 patients with early-stage breast cancer who underwent neoadjuvant chemotherapy followed by curative surgery between January 2006 and December 2022. Clinicopathological data, including age at diagnosis, clinical T stage, clinical node positivity, estrogen receptor (ER) status, progesterone receptor (PR) status, HER2 status, and pCR, were collected. Baseline radiologic (ultrasound or MRI) findings according to the anatomical stage based on the 8th American Joint Committee on Cancer guidelines were used to assess clinical T stage and nodal status. Immunohistochemistry (IHC) of pre-treatment core biopsy samples was used to evaluate ER, PR, and HER2 status. Hormone receptor (HR) was considered positive if either ER or PR was positive (Allred scores of 3–8 or ≥ 1 % of stained cancer cell nuclei). HER2 status was defined as positive for IHC scores of 3+ or 2+ with gene amplification by fluorescent in situ hybridization analysis, and negative for IHC scores of 0 or 1+, according to the ASCO-CAP guidelines [14]. pCR was defined as the absence of invasive tumor cells in both the breast and axilla (ypT0/is, ypN0) on pathological evaluation of the surgical specimen after neoadjuvant chemotherapy.

Among all patients, those with suspected intracranial lesions on radiological evaluation or confirmed by pathological examination were classified as having BM. In this cohort, we assessed the date of diagnosis of brain metastasis whether brain metastasis represented the first recurrence event, and the presence of extracranial metastases at the time of diagnosis. Additionally, data regarding whole-brain radiotherapy (WBRT), Karnofsky Performance status, and craniotomy performance were collected.

### 2.2 Gene expression datasets and analysis

Nine samples were obtained from patients with BM who underwent craniotomy. To obtain gene expression profiles using microarrays, total RNA was extracted from each tissue sample using the Qiagen RNeasy Plus Mini kit. The collected RNA was loaded onto an Illumina HumanHT-12 v4 Expression BeadChip (Illumina, San Diego, CA, USA). Variance-stabilizing transformation and quantile normalization of data using the R/Bioconductor *lumi* package were performed. Differentially expressed genes (DEGs) were calculated using the *limma* package, with grouping based on the pCR status. Volcano plots were generated using the *EnhancedVolcano* package. Genes were functionally annotated via overrepresentation analysis using GO gene sets and visualized as a dot plot using the *clusterProfiler* package. The enriched GO terms were stratified according to their kappa scores (>0.4). An enrichment plot was generated using GenePattern 2.0.

### 2.3 Statistical analysis

According to BM and pCR status, continuous values were analyzed using Student's t-test, and categorical values were compared using chi-square or Fisher's exact tests. To elucidate the BM characteristics independently associated with pCR, a binary Cox regression model was used, adjusted for other related variables such as age (continuous value), clinical tumor stage (2 vs. ≥ 3), clinical nodal stage (0–1 vs. 2–3), and subtypes (HR + HER2-vs. HR + HER2+ vs. HR-HER2+ vs. TNBC).

The brain metastasis-free interval (BMFI) was defined as the period from the date of breast cancer diagnosis to the recurrence of the intracranial lesion. Intracranial progression-free survival (iPFS) was defined as the period from the date of brain metastasis to the progression of the intracranial lesion. Overall survival (OS) was defined as the period from the date of brain metastasis to death from any cause or the last censored day. The Kaplan–Meier method was used to estimate the BMFI, iPFS, and OS rates, and the results of the groups were compared using the log-rank test.

In the BM cohort, one-to-one propensity score matching (PSM) was performed using the nearest-neighbor matching method with a caliper width of 0.2 standard deviations of the logit distance measured using the R package, “*MatchIt*.” Clinicopathological factors, including age at diagnosis, subtype, number of metastatic lesions, brain metastasis as 1st recurrence event, extracranial metastasis, and Karnofsky Performance Status, were included in the PSM.

All statistical analyses were performed using SPSS version 27 (SPSS: Chicago, IL, USA) and R version 4.1.3 (R Foundation for Statistical Computing, Vienna, Austria). Statistical significance was set at *P* < 0.05.

## 3 Results

### 3.1 Study population

**Supplementary Fig. 2:**
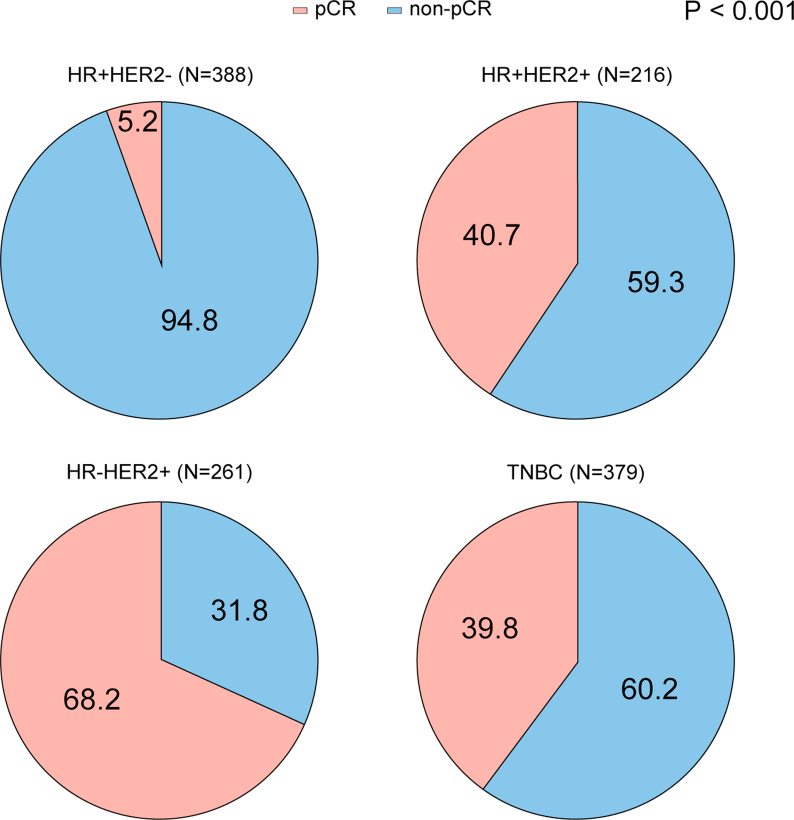
Pathologic complete response (pCR) rates according to breast cancer subtypes. HR, hormone receptor; HER2, human epidermal growth factor receptor 2; TNBC, triple-negative breast cancer.

We analyzed 1,244 patients with early-stage breast cancer who underwent neoadjuvant chemotherapy. The patients’ median age was 48 years (interquartile range, 42–56 years), and most patients were at clinical stage 2 or higher or clinically node-positive (Table 1). A total of 437 patients (35.1 %) achieved pCR. The pCR rates by subtype were 5.2 % in HR + HER2-, 40.7 % in HR + HER2+, 68.2 % in HR-HER2+, and 39.8 % in TNBC (*P* < 0.001, Supplementary Fig. 2). The majority of patients with HR + HER2-breast cancer received anthracycline and taxane-based neoadjuvant chemotherapy. Among those with HER2-positive breast cancer, 86 % received HER2-targeted therapy, with dual HER2-blockade in approximately 80 %. In TNBC, only five patients received the pembrolizumab-based neoadjuvant chemotherapy; 66.5 % received anthracycline and taxane-based regimen and 32.2 % also received carboplatin (Supplementary Table S1).

**Table 1:** Characteristics of patients who underwent neoadjuvant chemotherapy for brain metastasis.

Variables	Non-brain metastasis (N = 1,192)	Brain metastasis (N = 52)	Total (N = 1,244)	*P*-value
Age (median, IQR)	48 (42–56)	47.5 (41.25–55.5)	48 (42–56)	0.565
Clinical tumor stage				0.163∗
1	78 (6.5)	0	78 (6.3)	
2	696 (58.4)	34 (65.4)	730 (58.7)	
≥3	418 (35.1)	18 (34.6)	436 (35.0)	
Clinical nodal stage				<0.001
0	187 (15.7)	1 (1.9)	188 (15.1)	
1	510 (42.8)	13 (25.0)	523 (42.0)	
2	444 (37.2)	30 (57.7)	474 (38.0)	
3	51 (4.3)	8 (15.4)	59 (4.7)	
Subtypes				0.006
HR + HER2-	381 (32.0)	7 (13.5)	388 (31.2)	
HR + HER2+	208 (17.4)	8 (15.4)	216 (17.4)	
HR-HER2+	250 (21.0)	11 (21.2)	261 (21.0)	
TNBC	353 (29.6)	26 (50.0)	379 (30.5)	
pCR				0.205
No	769 (64.5)	38 (73.1)	807 (64.9)	
Yes	423 (35.5)	14 (26.9)	437 (35.1)	

Note: Unless otherwise noted, the values represent the number of patients with percentages in parentheses.

*P*-values were obtained using the Fisher's exact test.

IQR, interquartile range; HR, hormone receptor; HER2, human epidermal growth factor receptor 2; TNBC, triple-negative breast cancer; pCR, pathological complete response.

**Fig. 1:**
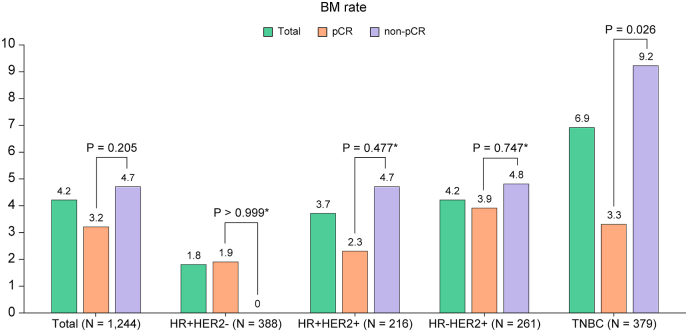
**Brain metastasis rate according to breast cancer subtypes stratified by pathologic complete response (pCR)**. HR, hormone receptor; HER2, human epidermal growth factor receptor 2; TNBC, triple-negative breast cancer.

**Supplementary Fig. 3:**
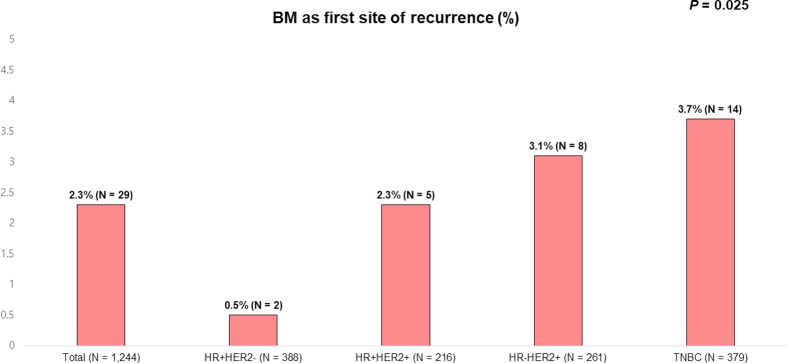
The rate of brain metastasis as first site of recurrence according to breast cancer subtypes. HR, hormone receptor; HER2, human epidermal growth factor receptor 2; TNBC, triple-negative breast cancer.

Overall, 52 patients (4.2 %) experienced BM. Patients with BM had a higher clinical nodal stage (clinical node-positive rate: 98.1 % vs. 84.3 %, *P* < 0.001) and TNBC proportion (50.0 % vs. 29.6 %, *P* = 0.006) than those without brain metastases. The pCR rate was lower in patients with BM (26.9 % vs. 35.5 %, *P* = 0.205); however, this difference was not significant (Table 1). The incidence of BM by subtype was 1.8 % in HR + HER2-, 3.7 % in HR + HER2+, 4.2 % in HR-HER2+, and 6.7 % in TNBC (Fig. 1). Similarly, the reate of BM as the first site of recurrence was lowest in HR + HER2-with a stepwise increase toward TNBC (Supplementary Fig. 3). Notably, in TNBC, the non-pCR group had a significantly higher rate of BM than the pCR group (9.2 % vs. 3.3 %, *P* = 0.026), a pattern absent in the other subtypes (Fig. 1).

### 3.2 Characteristics of brain metastasis by treatment response

**Supplementary Fig. 4:**
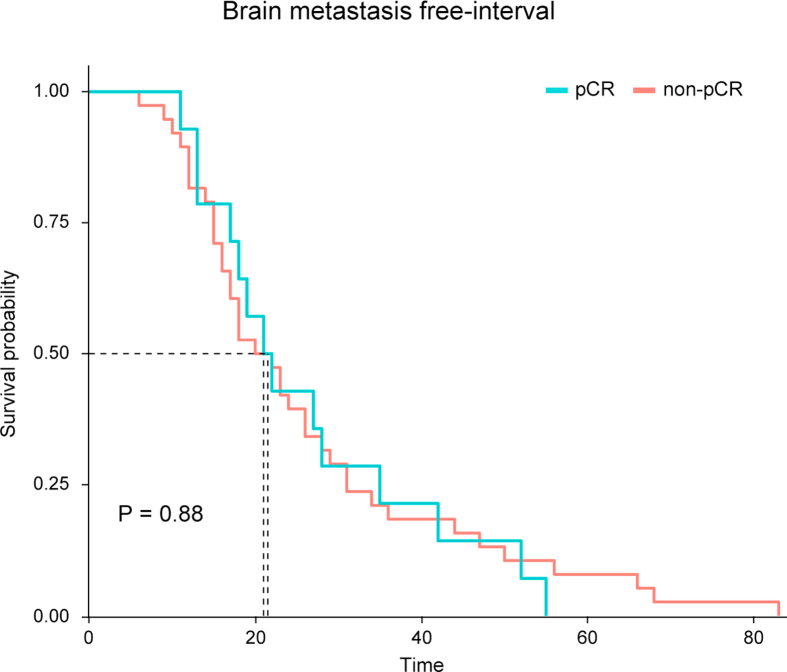
Brain metastasis-free interval according to pathologic complete response (pCR). pCR, pathologic complete response.

Among the patients with BM, 38 (73.1 %) had residual invasive disease after neoadjuvant chemotherapy, while 14 (26.9 %) achieved pCR (Table 2). Additionally, BM as the first recurrence event was more frequent in the pCR cohort (78.6 % vs. 47.4 %, *P* = 0.044). The median BMFI was 20 and 21 months in the non-pCR and pCR groups, respectively, with no significant difference between the two groups (*P* = 0.877, Supplementary Fig. 4).

**Table 2:** Characteristics according to pCR status in patients with brain metastasis.

Variables	Non-pCR (n = 38)	pCR (n = 14)	*P*-value
Age	46 (26–67)	52 (36–64)	0.260
Clinical tumor stage			>0.999∗
2	25 (65.8)	9 (64.3)	
≥3	13 (34.2)	5 (35.7)	
Clinical nodal stage			0.472∗
0-1	11 (28.9)	2 (14.3)	
2-3	27 (71.1)	12 (85.7)	
Subtypes			0.014∗
HR + HER2-	7 (18.4)	0	
HR + HER2+	6 (15.8)	2 (14.3)	
HR-HER2+	4 (10.5)	7 (50.0)	
TNBC	21 (55.3)	5 (35.7)	
Craniotomy			0.010
No	26 (68.4)	4 (28.6)	
Yes	12 (31.6)	10 (71.4)	
WBRT			0.254∗
No	9 (23.7)	1 (7.1)	
Yes	29 (76.3)	13 (92.9)	
No. of brain metastatic lesion			0.016∗
1	4 (10.5)	6 (42.9)	
≥2	34 (89.5)	8 (57.1)	
Brain metastasis as 1st recurrence			0.044
No	20 (52.6)	3 (21.4)	
Yes	18 (47.4)	11 (78.6)	
Extracranial metastasis			0.003
No	10 (26.3)	10 (71.4)	
Yes	28 (73.7)	4 (28.6)	
Karnofsky Performance Status			0.813∗
>80	17 (44.7)	5 (35.7)	
70-80	17 (44.7)	8 (57.1)	
<70	4 (10.5)	1 (7.1)	

∗*P*-value was obtained using Fisher's exact test.

pCR, pathologic complete response; HR, hormone receptor; HER2, human epidermal growth factor receptor 2; TNBC, triple-negative breast cancer; WBRT, whole-brain radiotherapy.

Compared with those in the non-pCR group, patients in the pCR group had a higher proportion of craniotomy procedures (71.4 % vs. 31.6 %, *P* = 0.010), single brain lesions (42.9 % vs. 10.5 %, *P* = 0.016), and BM as the first recurrence event (78.6 % vs. 47.4 %, *P* = 0.044). In contrast, the proportion of patients with extracranial metastases at the time of BM diagnosis (28.6 % vs. 73.7 %, *P* = 0.003) was lower in the pCR group (Table 2). Particularly, in the multivariable analysis adjusted for other relevant factors, the multiple metastatic brain lesions (odds ratio [OR], 0.11; 95 % confidential interval [CI], 0.01–0.92; *P* = 0.041) and extracranial metastases (OR, 0.11; 95 % CI, 0.02–0.74; *P* = 0.023) were significantly associated with pCR (Table 3).

**Table 3:** Relationship between pCR and characteristics of brain metastasis.

Features of brain metastasis	pCR	Adjusted OR^a^	95 % CI	*P*-value
Multiple brain metastasis (≥2 sites)	non-pCR	Ref		
	pCR	0.11	0.01-0.92	0.041
Late recurrence (≥2nd line)	non-pCR	Ref		
	pCR	0.28	0.05-1.49	0.136
Extracranial metastasis	non-pCR	Ref		
	pCR	0.11	0.02-0.74	0.023

pCR, pathologic complete response; OR, odds ratio; CI, confidence interval.

a Adjusted for age (continuous value), cT stage (2 vs. ≥ 3), cN stage (0–1 vs. 2–3), subtypes (HR + HER2-vs. HR + HER2+ vs. HR-HER2+ vs. TNBC).

### 3.3 Prognosis in patients with brain metastasis

**Fig. 2:**
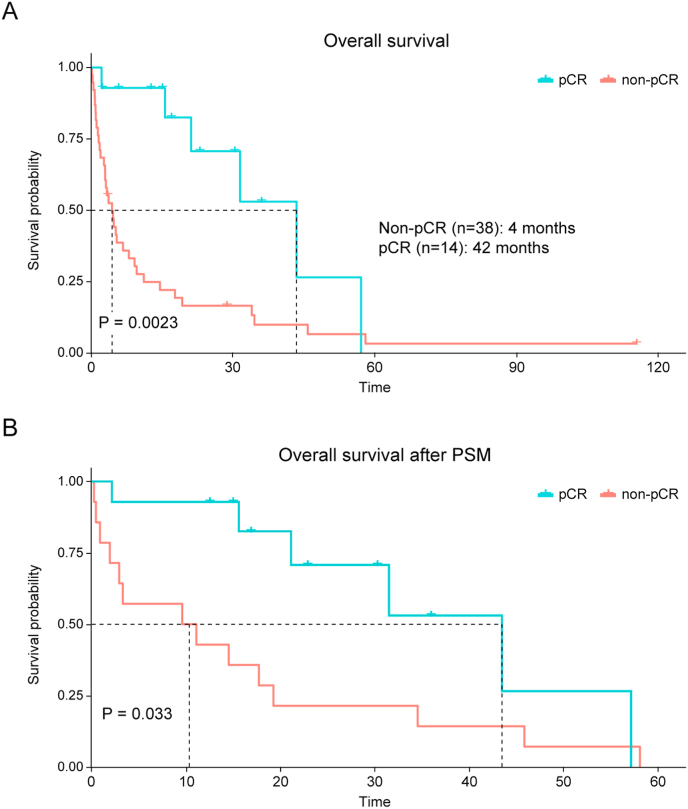
Overall survival according to pathologic complete response (pCR) in (A) all patients with brain metastasis and (B) a propensity score-matched cohort.

**Supplementary Fig. 5:**
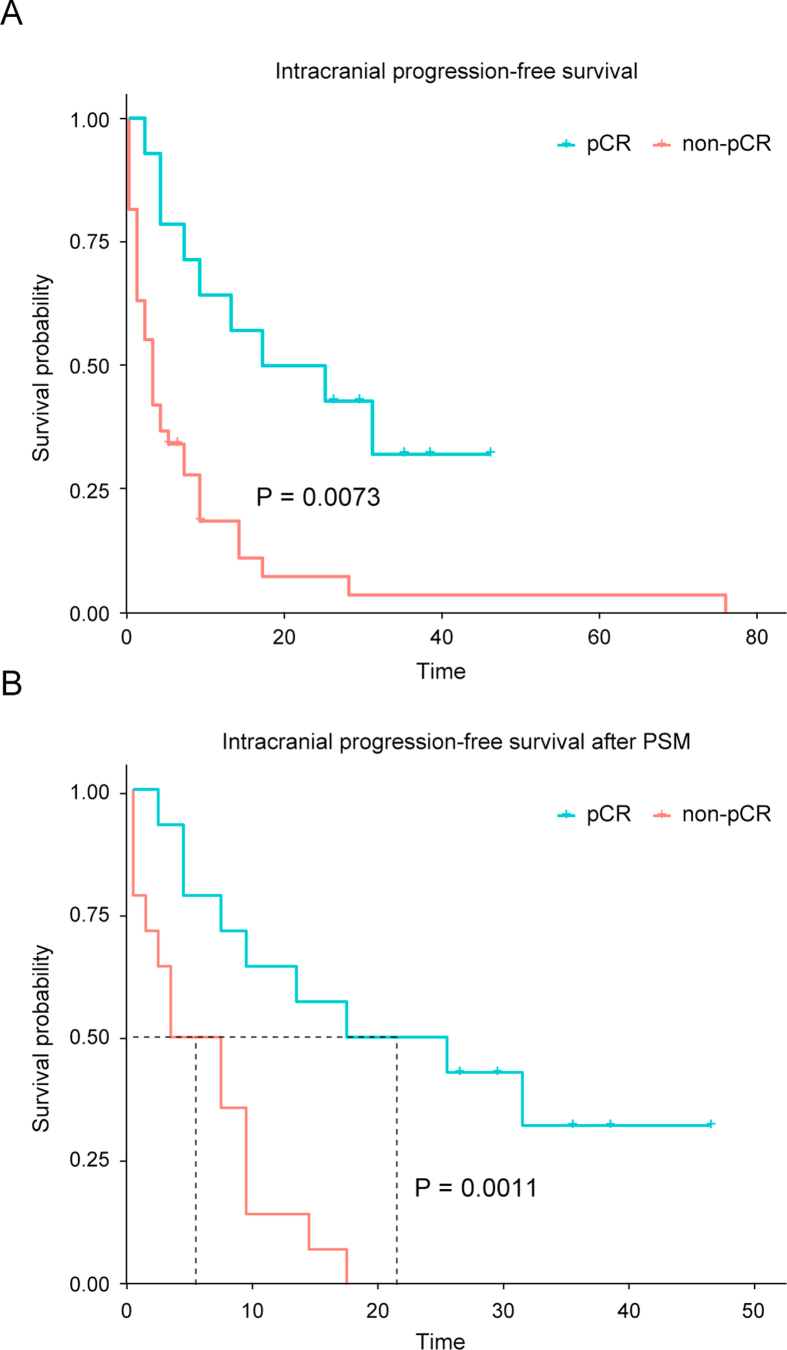
Intracranial progression-free survival according to pathologic complete response (pCR) in (A) all patients with brain metastasis (BM) and (B) a propensity score-matched cohort. pCR, pathologic complete response.

**Supplementary Fig. 6:**
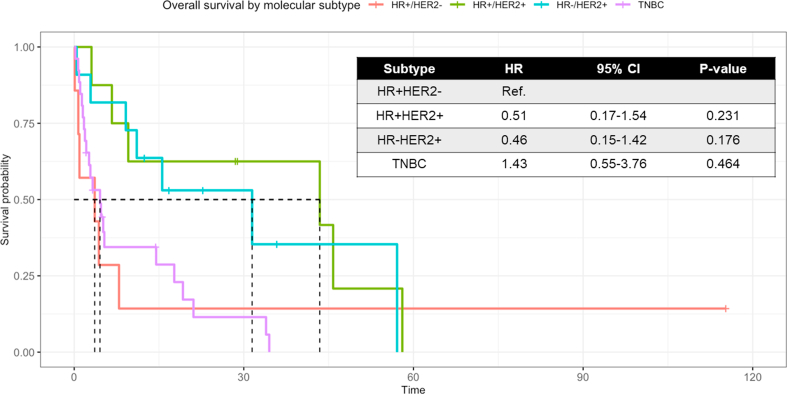
Overall survival according to breast cancer subtypes. HR, hormone receptor; HER2, human epidermal growth factor receptor 2; TNBC, triple-negative breast cancer.

In the BM cohort, survival outcomes differed significantly depending on treatment response to neoadjuvant chemotherapy. iPFS was 17 and 3 months in the pCR and non-pCR groups, respectively (*P* = 0.001, Supplementary Fig. 5A). During the median follow up of 5.5 months, the median OS from the time of BM diagnosis was 42 and 4 months in the pCR and non-pCR groups, respectively (*P* = 0.002; Fig. 2A). Furthermore, OS tended to be longer in HER2-positive breast cancer, but the difference did not reach statistical significance (Supplementary Fig. 6).

PSM was performed to adjust for baseline differences between patients who achieved pCR with a more favorable prognostic potential than those with invasive residual disease after neoadjuvant chemotherapy. After matching, the characteristics of the two groups were well balanced (Supplementary Table 2). In the PSM cohort, the pCR group continued to exhibit superior iPFS (median iPFS: 21 months in the pCR group vs. 5 months in the non-pCR group, *P* = 0.001; Supplementary Fig. 5B) and OS (median OS: 43 months in pCR group vs. 10 months in the non-pCR group, *P* = 0.033; Fig. 2B).

### 3.4 Gene expression in patients with brain metastasis

**Fig. 3:**
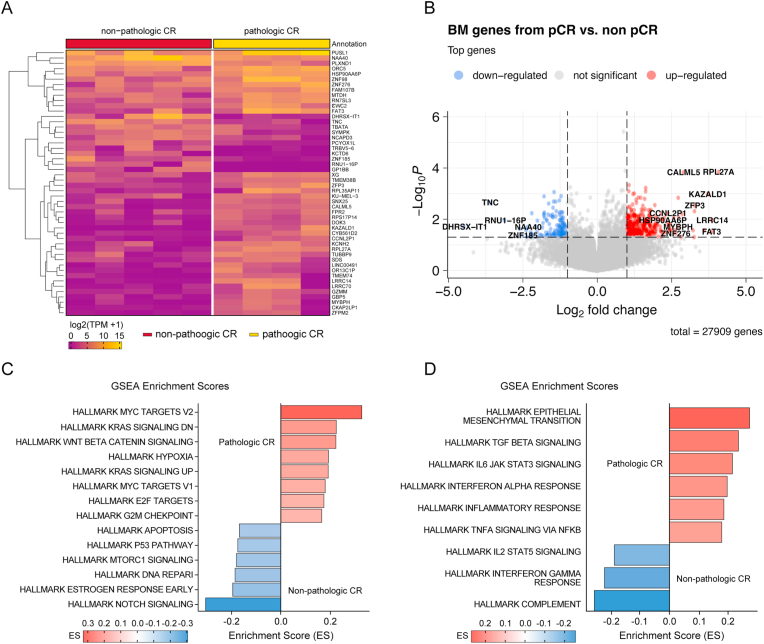
**Molecular profiling of brain metastases based on pathologic complete response (pCR) status.** (A) Heatmap of differentially expressed genes (DEGs) in brain metastasis (BM) samples from breast cancer patients with and without pathologic complete response (pCR) following neoadjuvant chemotherapy. (B) Volcano plot illustrating the distribution of DEGs between pCR and non-pCR BM samples. (C) Gene Ontology (GO) enrichment analysis of genes upregulated in the pCR group. (Oncologic pathway) (D) GO analysis of genes upregulated in the pCR groups (Immune related pathway).

**Supplementary Fig. 7:**
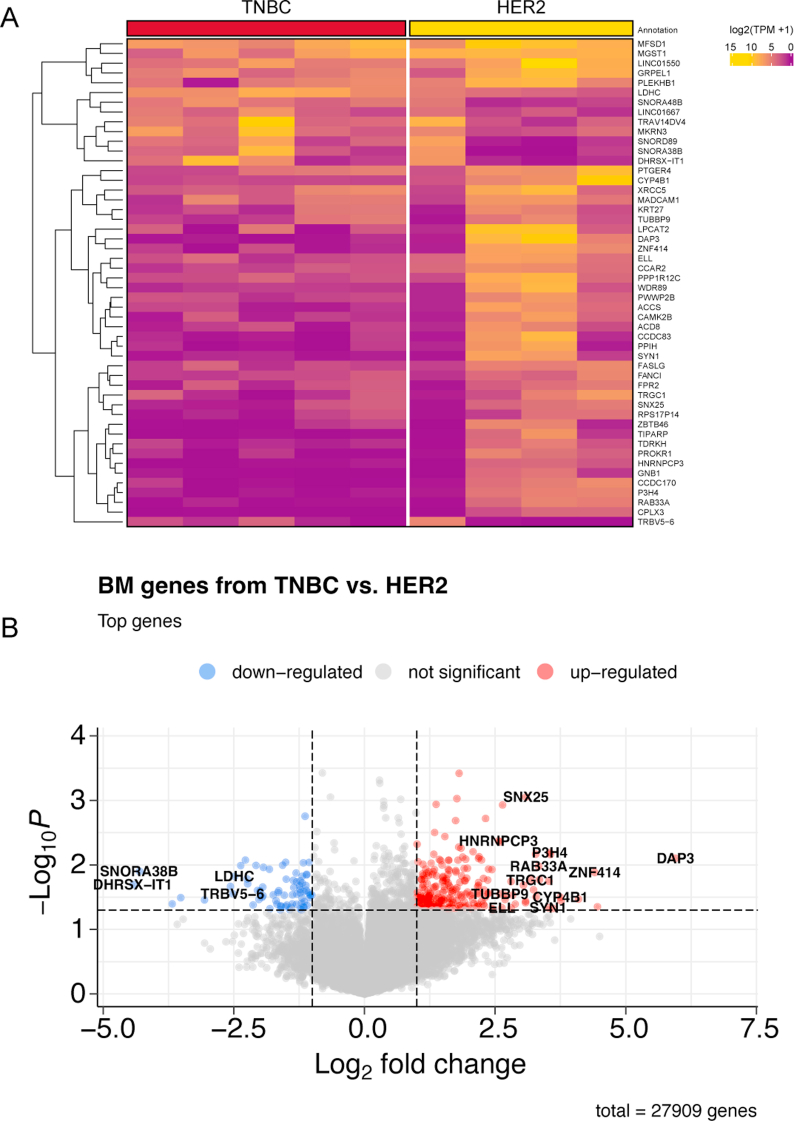
Differentially expressed genes (DEGs) Between HER2+ and TNBC Patients: Heatmap (A) and volcano plot (B). HER2, human epidermal growth factor receptor 2; TNBC, triple-negative breast cancer.

**Supplementary Fig. 8:**
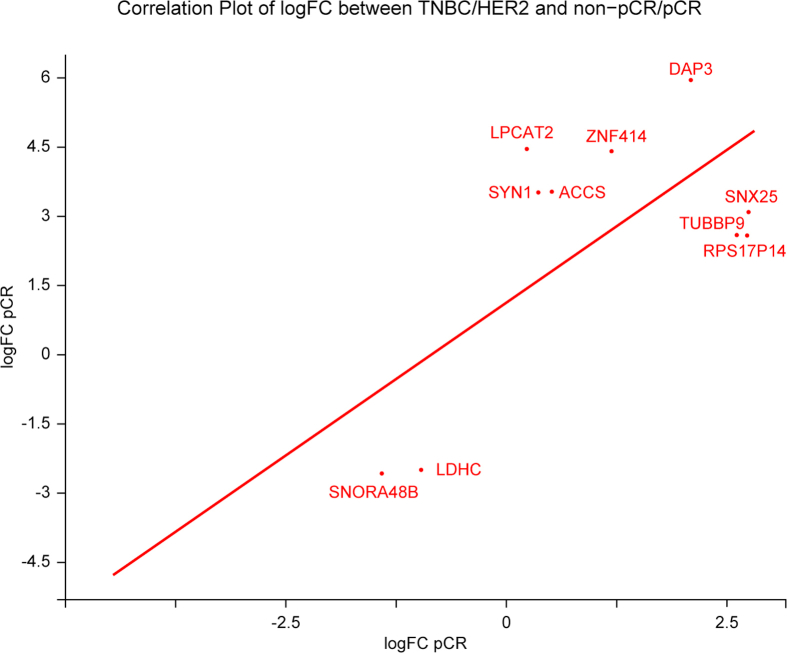
Correlation analysis between differentially expressed genes (DEGs) in HER2+ vs. TNBC and DEGs in pathologic complete response (pCR) vs. non-pCR. HER2, human epidermal growth factor receptor 2; TNBC, triple-negative breast cancer.

In this cohort, we analyzed nine patients with BM, comprising five non-pCR patients and four with pCR. Among the five non-pCR patients, four had TNBC and one had HER2-positive breast cancer. Among the four patients with pCR, three had HER2-positive breast cancer and one had TNBC (Fig. 3A and B). Overall, 186 DEGs were identified. In the pCR group, 77 upregulated DEGs were identified, including *RPL27A*, *CTLA4*, *CD82*, *GPR183*, *MEIS1*, *EXOC7*, *DOK3*, *RASSF6*, *SORCS2*, *POU2F1*, and *IL1*7RE. In the non-pCR group, 28 upregulated DEGs were identified, including *PLXND1*, *TNC (Tenascin-C)*, *CITED1*, *EGFL6*, *FLRT1*, *PNKP*, *UROD*, and *MARCHF6*. The results of the analysis based on the TNBC and HER2 subtypes are presented in Supplementary Fig. 7 as a volcano plot. Given the small sample size, there may have been overlapping genes between the non-pCR vs. pCR and TNBC vs. HER2 groups. To differentiate between these effects, correlation analysis was conducted, identifying *SNX25*, *TUBBP9*, and *RPS17P14* as genes associated with pCR, while *LDHC* and *SNORA48B* were associated with non-pCR (Supplementary Fig. 8). Gene Set Enrichment Analysis of 50 hallmark gene sets revealed increased activity of *MYC targets V1* and *V2*, *KRAS* signaling, *beta-catenin* signaling, and *hypoxia* pathways in the pCR group. In the non-pCR group, upregulation of *NOTCH*, *MTORC1*, *p53*, and *DNA repair*-related pathways was observed (Fig. 3C). Regarding immune-related hallmark pathways, the pCR group showed increased activity in *epithelial-mesenchymal transition*, *interferon-alpha response*, *TGF-beta*, and *IL6-JAK-STAT3* signaling. In contrast, the non-pCR group exhibited upregulation of *IL2-STAT5* signaling, *interferon-gamma response*, and *complement* pathway (Fig. 3D).

## 4 Discussion

In this study, the effect of the treatment response to neoadjuvant chemotherapy on BM patterns and survival outcomes was investigated. Among 1,244 patients who received neoadjuvant chemotherapy, 52 (4.2 %) developed BM. One of the key findings of our study was the differential incidence of BM based on breast cancer subtypes stratified by treatment response. Consistent with previous studies [15,16], we observed that patients with TNBC exhibited the highest brain metastasis rate (6.7 %), followed by HER2-positive subtypes (4.2 % in HR-HER2+ and 3.7 % in HR + HER2+); however, HR + HER2-had the lowest incidence (1.8 %). Notably, in HER2-positive breast cancer, BM rates did not significantly differ between the pCR and non-pCR groups, consistent with data from the Memorial Sloan Kettering Cancer Center involving patients treated with trastuzumab- and pertuzumab-based neoadjuvant systemic therapy [17]. In contrast, among patients with TNBC, those with residual invasive disease had a significantly higher BM rate than those who achieved pCR (9.2 % vs. 3.3 %), suggesting that achieving pCR may mitigate the risk of BM in TNBC and highlighting the importance of effective neoadjuvant chemotherapy for this aggressive subtype.

We also analyzed BM characteristics based on the treatment response to neoadjuvant chemotherapy. Patients with residual invasive disease (non-pCR) are more likely to have multiple brain lesions and extracranial metastases at the time of BM diagnosis. Moreover, prognosis differed significantly according to the pCR status; the median iPFS was notably longer in the pCR group (17 months vs. 3 months), and the median OS from the time of BM diagnosis was markedly prolonged (42 months vs. 4 months). The difference in survival outcomes between the two groups remained significant after PSM was performed to balance the baseline characteristics. These findings indicate that patients who achieve pCR can still develop brain metastases; however, their metastatic burden tends to be more localized and may be more amenable to aggressive local treatment strategies, including surgical resection or brain radiotherapy.

Previous studies have revealed that patients with brain oligometastases (those with four or fewer metastatic lesions) have a more favorable prognosis than those with extensive metastases [18]. Surgical resection with or without radiotherapy has been associated with survival benefits in patients with a single brain lesion [[19], [20], [21], [22]]. Among the patients in the pCR group in our study, 43 % had a single brain metastasis, and approximately 70 % underwent craniotomy. Conversely, the extracranial disease, more frequently observed in the non-pCR group, was associated with a significantly worse prognosis, despite the active application of local therapy [21,22]. In addition to these clinical features, our gene expression analysis revealed upregulation of NOTCH, MTORC1, p53, and DNA repair-related pathways in the non-pCR group, suggesting that these findings may be associated with a more aggressive tumor biology. Collectively, these findings underscore the need for prompt and intensive management strategies in the non-CR patients with BM and highlight the prognostic value of pCR status in predicting intracranial disease behavior.

Despite the distinct BM characteristics based on treatment response, there was no significant difference in the period from the initial diagnosis to the development of BM between the pCR and non-pCR groups. This finding may be explained by several factors. Considering the low incidence of BM as the first site of recurrence in the non-pCR cohort, these patients frequently experience early extracranial progression and shorter survival. These competing-risks increase censoring and may attenuate differences in observed BMFI. Furthermore, patients who achieve pCR but subsequently develop BM may constitute a biologically selected subgroup with intrinsically aggressive metastatic potential, thereby exhibiting a similar interval to intracranial relapse. These observations suggest that treatment response primarily affects the likelihood—not the temporal pattern—of BM development.

Recently, active surveillance for BM has been considered, even in asymptomatic patients with HER2-positive breast cancer and TNBC [7,23]. However, our findings suggest that active screening eligibility criteria are not sufficiently refined by the treatment response to neoadjuvant chemotherapy. Nevertheless, active screening may be necessary for patients in the pCR group who develop BM, have a lower tumor burden, and are expected to achieve significantly improved survival outcomes with aggressive treatment. Additionally, emerging technologies for minimal residual disease assessment, such as circulating tumor DNA or tumor cell detection, are expected to facilitate early detection of BM, particularly in the excellent treatment response subpopulation [24,25].

Although accumulating evidence has linked well‒established clinicopathological factors—younger age, higher clinical, pathologic stage, tumor grade, or Ki-67 expression—to BM risk [15,26], the specific molecular mechanisms underlying BM development based on treatment response remain unclear. *RPL27A* is significantly upregulated in TNBC BM, through the activation of EIF2 signaling, thereby promoting tumor progression. However, this upregulation may be a TNBC effect rather than a consequence of pCR [27]. Similarly, CTLA4 expression varies according to the breast cancer subtype, with higher levels in HER2-positive and TNBC BM, suggesting a potential role in immune checkpoint inhibitor therapy. Notably, high *CTLA4* expression may be related to patients with TNBC and HER2 achieving pCR, indicating a possible link between immune modulation and treatment response [28]. *PLXND1*, a key target in tumor-associated endothelial cells, is elevated in these cells but shows reduced expression in patients with pCR, suggesting a potential prognostic role; however, paradoxically, it may also indicate the reduced efficacy of *PLXND1*--targeted therapy in the pCR group [29]. Based on these findings, further investigation into the molecular pathways driving breast cancer BM is necessary, particularly in relation to treatment responses and immune modulation.

This study has some limitations. First, the retrospective nature of our analysis introduced a potential selection bias, although PSM was performed to balance the baseline characteristics of patients with BM, unmeasured confounders might have influenced the observed survival differences. Second, despite the large overall cohort, the absolute number of patients who subsequently developed brain metastases was relatively small (4.2 %), inherently limiting the statistical power of subgroup analyses. Third, the number of BM samples available for transcriptomic profiling was limited. This reflects both the low incidence of BM after curative-intent neoadjuvant therapy and the practical difficulty of obtaining sufficient tissue from all affected patients. Because this study was not prospectively designed, corresponding primary biopsy samples were note collected for comparative analyses, which woud have further strengthened the translational insights into metastatic evolution. Finally, bulk RNA sequencing may not fully capture intratumoral heterogeneity; single-cell or spatial approaches could provide more granular information. Future prospective studies with larger cohorts integrating paired primary and intracranial tissue with high-resolution molecular profiling are warranted to validate these findings and further elucidate the mechanisms underlying BM development according to treatment response.

This study highlights different BM characteristics based on treatment response to neoadjuvant chemotherapy in patients with breast cancer. Patients who achieved pCR exhibited a lower BM burden and significantly improved survival. The molecular differences between patients with pCR and non-pCR with BM suggest distinct metastatic pathways with potential therapeutic implications. These findings also indicate that treatment response to neoadjuvant chemotherapy may serve as practical indicatior to tailor surveillance intensity and subsequent treatment. Future research should focus on refining surveillance strategies and exploring targeted therapies for patients with BM to mitigate high-risk metastatic profiles.

## CRediT authorship contribution statement

**Jihwan Yoo:** Writing – review & editing, Writing – original draft, Supervision, Resources, Methodology, Investigation, Funding acquisition, Formal analysis, Data curation, Conceptualization. **Yoon Jin Cha:** Writing – review & editing, Supervision, Methodology, Investigation, Funding acquisition, Formal analysis, Data curation. **Sung Gwe Ahn:** Supervision, Resources, Methodology, Investigation, Formal analysis, Data curation. **Joon Jeong:** Supervision, Resources, Methodology, Investigation, Formal analysis, Data curation. **Hun Ho Park:** Supervision, Resources, Methodology, Investigation, Formal analysis, Data curation. **Sung Jun Ahn:** Supervision, Resources, Methodology, Investigation, Formal analysis, Data curation. **Bio Joo:** Supervision, Resources, Methodology, Investigation, Formal analysis, Data curation. **Ji Hyun Park:** Supervision, Resources, Methodology, Investigation, Formal analysis, Data curation. **Jee Hung Kim:** Writing – review & editing, Writing – original draft, Supervision, Resources, Methodology, Investigation, Funding acquisition, Formal analysis, Data curation, Conceptualization. **Soong June Bae:** Writing – review & editing, Writing – original draft, Supervision, Resources, Methodology, Investigation, Funding acquisition, Formal analysis, Data curation, Conceptualization.

## Funding

This research was funded by the 10.13039/501100003725National Research Foundation of Korea (NRF) grants funded by the Korean government [grant numbers: RS-2023-00246346, NRF-2021R1G1A1093596, and NRF-2022R1I1A1A01065696]. In addition, this study was supported by a new faculty research seed money grant (2025-32-0033) and a faculty research grant (6-2024-0055) from 10.13039/501100008005Yonsei University College of Medicine. The NRF and Yonsei University College of Medicine played no role in study design, data collection, analysis, interpretation of data, or the writing of this manuscript.

## Declaration of competing interest

All authors declare no financial or potential competing interests.

## Data Availability

The datasets analyzed during the current study are not publicly available, but are available from the corresponding author on reasonable request.

## References

[1] Kennecke H., Yerushalmi R., Woods R., Cheang M.C., Voduc D., Speers C.H. (2010). Metastatic behavior of breast cancer subtypes. J Clin Oncol.

[2] Cho S., Joo B., Park M., Ahn S.J., Suh S.H., Park Y.W. (2023). A radiomics-based model for potentially more accurate identification of subtypes of breast cancer brain metastases. Yonsei Med J.

[3] Arvold N.D., Oh K.S., Niemierko A., Taghian A.G., Lin N.U., Abi-Raad R.F. (2012). Brain metastases after breast-conserving therapy and systemic therapy: incidence and characteristics by biologic subtype. Breast Cancer Res Treat.

[4] Morrison L., Loibl S., Turner N.C. (2024). The CDK4/6 inhibitor revolution - a game-changing era for breast cancer treatment. Nat Rev Clin Oncol.

[5] Lloyd M.R., Jhaveri K., Kalinsky K., Bardia A., Wander S.A. (2024). Precision therapeutics and emerging strategies for HR-positive metastatic breast cancer. Nat Rev Clin Oncol.

[6] Marra A., Chandarlapaty S., Modi S. (2024). Management of patients with advanced-stage HER2-positive breast cancer: current evidence and future perspectives. Nat Rev Clin Oncol.

[7] Gennari A., André F., Barrios C.H., Cortés J., de Azambuja E., DeMichele A. (2021). ESMO clinical practice guideline for the diagnosis, staging and treatment of patients with metastatic breast cancer. Ann Oncol.

[8] Debien V., De Caluwé A., Wang X., Piccart-Gebhart M., Tuohy V.K., Romano E. (2023). Immunotherapy in breast cancer: an overview of current strategies and perspectives. NPJ Breast Cancer.

[9] Cortazar P., Zhang L., Untch M., Mehta K., Costantino J.P., Wolmark N. (2014). Pathological complete response and long-term clinical benefit in breast cancer: the CTNeoBC pooled analysis. Lancet.

[10] Korde L.A., Somerfield M.R., Carey L.A., Crews J.R., Denduluri N., Hwang E.S. (2021). Neoadjuvant chemotherapy, endocrine therapy, and targeted therapy for breast cancer: ASCO guideline. J Clin Oncol.

[11] Litton J.K., Regan M.M., Pusztai L., Rugo H.S., Tolaney S.M., Garrett-Mayer E. (2023). Standardized definitions for efficacy end points in neoadjuvant breast cancer clinical trials: NeoSTEEP. J Clin Oncol.

[12] Yau C., Osdoit M., van der Noordaa M., Shad S., Wei J., de Croze D. (2022). Residual cancer burden after neoadjuvant chemotherapy and long-term survival outcomes in breast cancer: a multicentre pooled analysis of 5161 patients. Lancet Oncol.

[13] Galli G., Tessari A., Porcu L., Bregni G., Paolini B., Carcangiu M.L. (2017). Complete remission in metastatic breast cancer: expecting the unexpected-results of a cross-sectional study. Breast Cancer.

[14] Wolff A.C., Somerfield M.R., Dowsett M., Hammond M.E.H., Hayes D.F., McShane L.M. (2023). Human epidermal growth factor receptor 2 testing in breast cancer: ASCO-college of American pathologists guideline update. J Clin Oncol.

[15] Azim H.A., Abdel-Malek R., Kassem L. (2018). Predicting brain metastasis in breast cancer patients: stage versus biology. Clin Breast Cancer.

[16] Aversa C., Rossi V., Geuna E., Martinello R., Milani A., Redana S. (2014). Metastatic breast cancer subtypes and central nervous system metastases. Breast.

[17] Ferraro E., Singh J., Patil S., Razavi P., Modi S., Chandarlapaty S. (2022). Incidence of brain metastases in patients with early HER2-positive breast cancer receiving neoadjuvant chemotherapy with trastuzumab and pertuzumab. NPJ Breast Cancer.

[18] Joo B., Kim J.H., Ahn S.G., Park M., Suh S.H., Ahn S.J. (2024). De novo versus recurrent metastatic breast cancer affects the extent of brain metastases. J Neuro Oncol.

[19] Patchell R.A., Tibbs P.A., Walsh J.W., Dempsey R.J., Maruyama Y., Kryscio R.J. (1990). A randomized trial of surgery in the treatment of single metastases to the brain. N Engl J Med.

[20] Vecht C.J., Haaxma-Reiche H., Noordijk E.M., Padberg G.W., Voormolen J.H., Hoekstra F.H. (1993). Treatment of single brain metastasis: radiotherapy alone or combined with neurosurgery?. Ann Neurol.

[21] Noordijk E.M., Vecht C.J., Haaxma-Reiche H., Padberg G.W., Voormolen J.H., Hoekstra F.H. (1994). The choice of treatment of single brain metastasis should be based on extracranial tumor activity and age. Int J Radiat Oncol Biol Phys.

[22] Mintz A.H., Kestle J., Rathbone M.P., Gaspar L., Hugenholtz H., Fisher B. (1996). A randomized trial to assess the efficacy of surgery in addition to radiotherapy in patients with a single cerebral metastasis. Cancer.

[23] Komorowski A.S., Warner E., MacKay H.J., Sahgal A., Pritchard K.I., Jerzak K.J. (2020). Incidence of brain metastases in nonmetastatic and metastatic breast cancer: is there a role for screening?. Clin Breast Cancer.

[24] Morganti S., Parsons H.A., Lin N.U., Grinshpun A. (2023). Liquid biopsy for brain metastases and leptomeningeal disease in patients with breast cancer. NPJ Breast Cancer.

[25] Park H.S., Han H.J., Lee S., Kim G.M., Park S., Choi Y.A. (2017). Detection of circulating tumor cells in breast cancer patients using Cytokeratin-19 real-time RT-PCR. Yonsei Med J.

[26] Koniali L., Hadjisavvas A., Constantinidou A., Christodoulou K., Christou Y., Demetriou C. (2020). Risk factors for breast cancer brain metastases: a systematic review. Oncotarget.

[27] Zhao W., Li X., Nian W., Wang J., Wang X., Sun L. (2021). Ribosome proteins represented by RPL27A mark the development and metastasis of triple-negative breast cancer in mouse and human. Front Cell Dev Biol.

[28] Patel L., Kolundzic N., Abedalthagafi M. (2025). Progress in personalized immunotherapy for patients with brain metastasis. npj Precis Oncol.

[29] Roodink I., Verrijp K., Raats J., Leenders W.P.J. (2009). Plexin D1 is ubiquitously expressed on tumor vessels and tumor cells in solid malignancies. BMC Cancer.

