# Supplementary material for: Impact of treatment response to neoadjuvant chemotherapy on brain metastasis patterns and breast cancer prognosis
Source: Breast. 2025 Nov 14;85:104650. doi: 10.1016/j.breast.2025.104650 (PMC12666593; doi:10.1016/j.breast.2025.104650)
Supplement: Multimedia component 1 [file mmc1.docx]

**Supplementary Table 1. Neoadjuvant chemotherapy regimens.**

| Neoadjuvant chemotherapy | N (%) |
| --- | --- |
| HR+HER2- |  |
| Anthracycline and taxane-based | 383 (98.7) |
| Non-anthracycline and taxane-based | 5 (1.3) |
| HER2+ |  |
| Anthracycline and taxane-based | 66 (13.8) |
| Chemotherapy plus trastuzumab | 34 (7.1) |
| Chemotherapy plus trastuzumab and pertuzumab | 377 (79.1) |
| TNBC |  |
| Anthracycline and taxane-based | 252 (66.5) |
| Anthracycline and taxane with carboplatin | 122 (32.2) |
| KEYNOTE-522 regimen | 5 (1.3) |

HR, hormone receptor; HER2, human epidermal growth factor receptor 2; TNBC, triple-negative breast cancer.

| **Supplementary Table 2. Characteristics according to pCR in propensity score matching cohort** | | | |
| --- | --- | --- | --- |
| Variables | Non-pCR (n = 14) | pCR (n = 14) | *P*-value |
| Age | 50.4+/-11.2 | 50.6+/-8.7 | 0.955 |
| Subtypes |  |  | > 0.999 |
| HR+HER2- | 0 | 0 |  |
| HR+HER2+ | 3 (21.4) | 2 (14.3) |  |
| HR-HER2+ | 3 (21.4) | 7 (50.0) |  |
| TNBC | 8 (57.1) | 5 (35.7) |  |
| Number of brain metastatic lesions |  |  | 0.209 |
| 1 | 2 (14.3) | 6 (42.9) |  |
| ≥ 2 | 12 (85.7) | 8 (57.1) |  |
| Brain metastasis as 1^st^ recurrence |  |  | > 0.999 |
| No | 3 (21.4) | 3 (21.4) |  |
| Yes | 11 (78.6) | 11 (78.6) |  |
| Extracranial metastasis |  |  | > 0.999 |
| No | 9 (64.3) | 10 (71.4) |  |
| Yes | 5 (35.7) | 4 (28.6) |  |
| Karnofsky Performance Status |  |  | 0.919 |
| > 80 | 4 (28.6) | 5 (35.7) |  |
| 70-80 | 9 (64.3) | 8 (57.1) |  |
| < 70 | 1 (7.1) | 1 (7.1) |  |

pCR, pathologic complete response; HR, hormone receptor; HER2, human epidermal growth factor receptor 2; TNBC, triple-negative breast cancer.
